# In vitro and in vivo silencing of plasmodial dhs and eIf-5a genes in a putative, non-canonical RNAi-related pathway

**DOI:** 10.1186/1471-2180-12-107

**Published:** 2012-06-13

**Authors:** Andreas Schwentke, Marcel Krepstakies, Ann-Kristin Mueller, Christiane Hammerschmidt-Kamper, Basma A Motaal, Tina Bernhard, Joachim Hauber, Annette Kaiser

**Affiliations:** 1 University Duisburg-Essen, Medical Research Centre, Institute of Pharmacogenetics, Hufelandstrasse 55, 45147, Essen, Germany; 2 Heinrich Pette Institute - Leibniz Institute for Experimental Virology, Martinistrasse 52, 20251, Hamburg, Germany; 3 Department of Infectious Diseases, Parasitology Unit, University Hospital Heidelberg, Im Neuenheimer Feld 324, 69120, Heidelberg, Germany

## Abstract

**Background:**

Deoxyhypusine synthase (DHS) catalyzes the first step in hypusine biosynthesis of eukaryotic initiation factor 5A (eIF-5A) in *Plasmodium falciparum*. Target evaluation of parasitic DHS has recently been performed with CNI-1493, a novel selective pro-inflammatory cytokine inhibitor used in clinical phase II for the treatment of Crohn's disease. CNI-1493 prevented infected mice from experimental cerebral malaria by decreasing the levels in hypusinated eIF-5A and serum TNF, implicating a link between cytokine signaling and the hypusine pathway.

Therefore we addressed the question whether either DHS itself or eIF-5A is required for the outcome of severe malaria. In a first set of experiments we performed an *in vitro* knockdown of the plasmodial eIF-5A and DHS proteins by RNA interference (RNAi) in 293 T cells. Secondly, transfection of siRNA constructs into murine *Plasmodium* schizonts was performed which, in turn, were used for infection.

**Results:**

293 T cells treated with plasmodial DHS- and eIF-5A specific siRNAs or control siRNAs were analyzed by RT-PCR to determine endogenous *dhs* -and *eIF-5A* mRNA levels. The expressed DHS-shRNA and EIF-5A-shRNA clearly downregulated the corresponding transcript in these cells. Interestingly, mice infected with transgenic schizonts expressing either the *eIF-5A* or *dhs* shRNA showed an elevated parasitemia within the first two days post infection which then decreased intermittently. These results were obtained without drug selection. Blood samples, which were taken from the infected mice at day 5 post infection with either the expressed EIF-5A-shRNA or the DHS-shRNA were analyzed by RT-PCR and Western blot techniques, demonstrating the absence of either the hypusinated form of eIF-5A or DHS.

**Conclusions:**

Infection of NMRI mice with schizonts from the lethal P. *berghei* ANKA wildtype strain transgenic for plasmodial eIF-5A-specific shRNA or DHS-specific shRNA resulted in low parasitemia 2–9 days post infection before animals succumbed to hyperparasitemia similar to infections with the related but non-lethal phenotype *P. berghei* strain NK65. RT-PCR and Western blot experiments performed with blood from the transfected erythrocytic stages showed that both genes are important for the proliferation of the parasite. Moreover, these experiments clearly demonstrate that the hypusine pathway in *Plasmodium* is linked to human iNos induction.

## Background

RNA interference (RNAi) is an evolutionary conserved mechanism found across a range of eukaryotes, where it plays a key role in post-transcriptional gene regulation and protection of genomes. The process of RNAi is triggered by the recognition of double-stranded RNA (dsRNA), which is then processed into 21–25 nucleotide sequences by Dicer, a cytoplasmic dsRNA specific RNaseII endonuclease [1]. The generated RNAs associate with an RNA-induced silencing complex (RISC) and unwind in a strand-specific manner [2]. The resulting short interfering RNAs (siRNAs) then target homologous mRNA for degradation in combination with the RNase H enzyme Argonaute (Slicer) [3]. The stage of double stranded (ds) RNA processing may be surpassed by experimentally introducing sequence-specific siRNAs directly into cells.

Given the immense Public Health costs for malaria disease and the need for new drug targets a silencing approach employing RNAi might be extremely beneficial for the development of novel and advanced therapeutic strategies. Moreover, the ability to use RNAi for gene silencing in *Plasmodium* would provide a powerful means to gain insight into pathogenic blood stages.

Recent experiments performed by molecular genetics suggested that RNAi is not functional in malaria parasites [4]. These authors showed that expression of the analyzed proteins continued despite the application of a variety of RNAi-based strategies to target genes which are non-essential to either growth or development of *P. falciparum* or *P. berghei*. In good agreement, control experiments with *Trypanosoma brucei*, a protozoan parasite with validated RNAi, were successful. Furthermore, to determine whether a primitive RNAi machinery exists in Apicomplexa a comparative analysis of Apicomplexan and other protozoan genomes was undertaken. Taken together these data argued that RNAi is absent in malaria parasites [4].

Several studies, however, reported the successful application of RNAi for gene silencing in the erythrocytic stages of *Plasmodium*. A series of experiments has been performed by introducing long dsRNAs by electroporation into infected erythrocytes. Gissot and coworkers [5] performed silencing experiments with MybB1, a transcription factor in *Plasmodium* thereby demonstrating its essential role in the erythrocytic stage. Kumar and colleagues [6] showed in a similar manner the requirement of a serine-threonine phosphatase for DNA-replication in *Plasmodium*. Tuteja and colleagues [7] identified a signal peptidase that is required for intra-erythrocytic growth by RNAi. Apart from electroporation [8], siRNAs have also been added directly to the culture medium. Cysteine proteases falcipain-1 and falcipain-2, which are necessary for haemoglobin degradation, have been shown to be essential for the blood stages [9]. However, this finding is in question since standard disruption techniques showed no effect on parasitic development in the blood stages [10]. While the latter authors suggested RNAi to be functional in *Plasmodium*, most of these cases resulted in parasitic death or significant growth defects due to unspecific downregulation of multiple genes by RNAi.

Deoxyhypusine synthase (DHS) catalyzes the first step in the biosynthesis of the amino acid hypusine (Hyp), a novel amino acid present in eukaryotic initiation factor 5A (eIF-5A) to form the deoxyhypusinylated intermediate. DHS transfers the aminobutyl moiety from the triamine spermidine to the є-amino group of Lys^50^ present in the hypusine loop. Both genes have been identified in *P. falciparum* and *P. vivax*[11,12]. Hitherto, the biological function of this posttranslational modification is unknown. Recent studies have implicated a permissive role of eIF-5A^Hyp^ in various diseases. In diabetes type 2 pancreatic stressed ß-cells [13] and in HIV-infected T cells, eIF-5A^Hyp^ is functional as a nucleocytoplasmic shuttle protein for the transport and translation of specific mRNAs [14].

Particularly in HIV, eIF-5A^Hyp^ is essential for the nucleocytoplasmic transport and translation of incompletely-spliced mRNAs encoding viral proteins [15,16]. In diabetes type2 eIF-5A^Hyp^ enables cytokine-mediated islet dysfunction through the direct posttranscriptional regulation of the mRNA encoding iNos2 (Nos2) in both rodent and human cells [13,17]. Importantly, the immunological events which lead to severe malaria are complex and parallel events present in HIV-infection and pancreatic stressed ß-cells. Exogenous NO administration [18,19] prevents the syndrome of severe malaria. Since a parasite specific nitric oxide synthase does not exist, the defense response may be attributed to the host specific iNos.

Cerebral malaria (CM) is characterized by clinical features like cognitive dysfunctions, seizures, coma and clinical parameters like anemia, metabolic acidosis, renal insufficiency and hypoglycaemia. Although the understanding of malaria pathogenesis is rudimentary, different theories have been accepted to understand the pathological process [20]. The sequestration theory suggests that seizures might be caused by the adherence of parasites to red blood cells and subsequent expression of parasite specific antigens which in turn lead to obstruction of blood flow, cerebral hypoxia and decreased removal of waste. For the neurological symptoms there is growing evidence that parasite-induced sequestration of infected and uninfected erythrocytes changes blood—brain barrier function. Moreover, host-specific immune mechanisms may be important in response to the presence of parasites in the CNS.

In a first step of the infection process parasitized red blood cells adhere to brain microvascular endothelial cells through the erythrocyte membrane protein [21]. After release of merozoites parasitic glycosylphosphatidylinositol (GPI) is released which induces a local inflammatory response involving natural killer and subsequently CD4^+^ T cells. At this stage of the infection, proinflammatory cytokines including tumor necrosis factor α (TNF-α interferon γ (IFN-γ and interleukin (IL)-1ß are produced locally before the entry of the systemic phase in which cytokines activate macrophages and CD8^+^ T cells [21]. In the systemic phase, more platelets and microparticles are released inducing perforin-mediated lesions in the endothelium [21].

Recently, metabolic changes in the central nervous system caused by the parasite, have been characterized as a third theory in explaining the pathology of malaria. During CM an increase of lactate and alanine concentration and alterations in tryptophane metabolites like the kynurenine pathway lead to an increased permeability of the blood brain barrier for plasma proteins.

DHS has been recently validated as a druggable target by the small molecule CNI-1493, a synthetic guanylhydrazone [22], which significantly extends the survival rate of *Plasmodium berghei* ANKA-infected C57BL/6 mice [22]. Initial studies with the compound suggested that the mechanism of action can be attributed to the inhibition of parasitic DHS and the translation of host specific TNFα-mRNA [23], indicating a link between host cell proinflammatory cytokine production and the hypusine pathway.

To study the outcome after an *in vivo* knockdown of this enzyme and its target protein eIF-5A in the erythrocytic stages of *Plasmodium* in more detail *,* we transfected siRNA constructs targeted to both genes based on *in vitro* knockdown experiments into *P. berghei* ANKA schizonts, using standard transfection methods [24].

## Results

### In vitro knock-down of P. falciparum DHS and eIF-5A by RNAi

**Figure 1:**
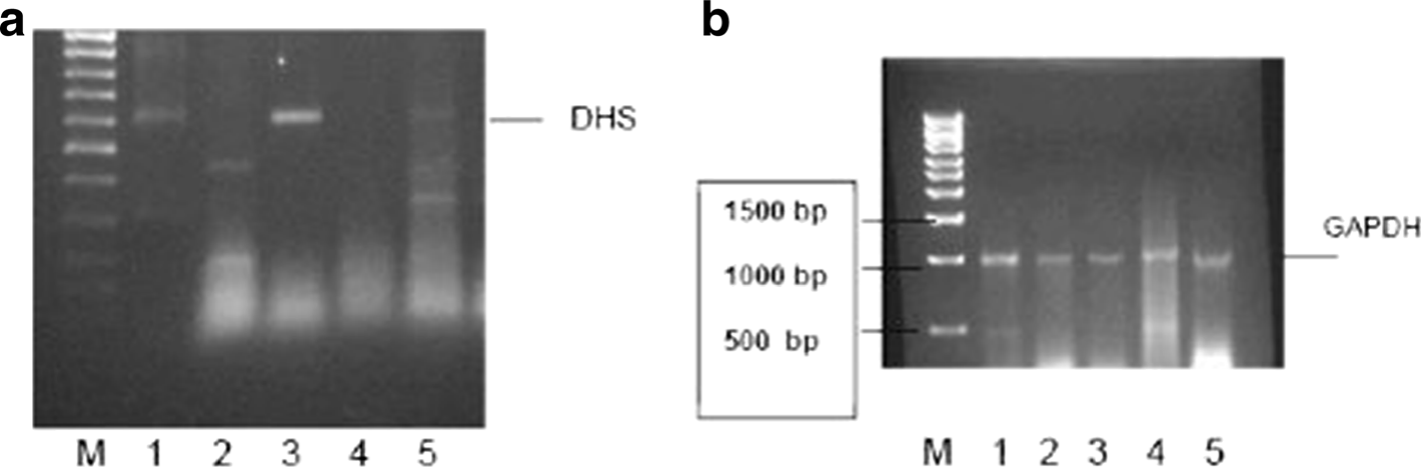
**A) Inhibition of plasmodial DHS by RNAi and monitoring of the 612 bp amplificate by RT-PCR after transfection of 293 T cells with the DHS expression vector.** 293T cells were cotransfected with: 1) Scramble II-duplex shRNA; 2) no transfected DNA; 3) the recombinant pcDNA3 vector containing 612 bp of a -highly conserved region of the *dhs* gene from *P. falciparum* (amino acid positions 208–412); 4) DHS- shRNA construct P#176; 5) DHS- shRNA construct P#43. **B**) Analysis of the 983 bp GAPDH amplificate in the cotransfected 293T cells described in Figure 1A.

**Figure 2:**
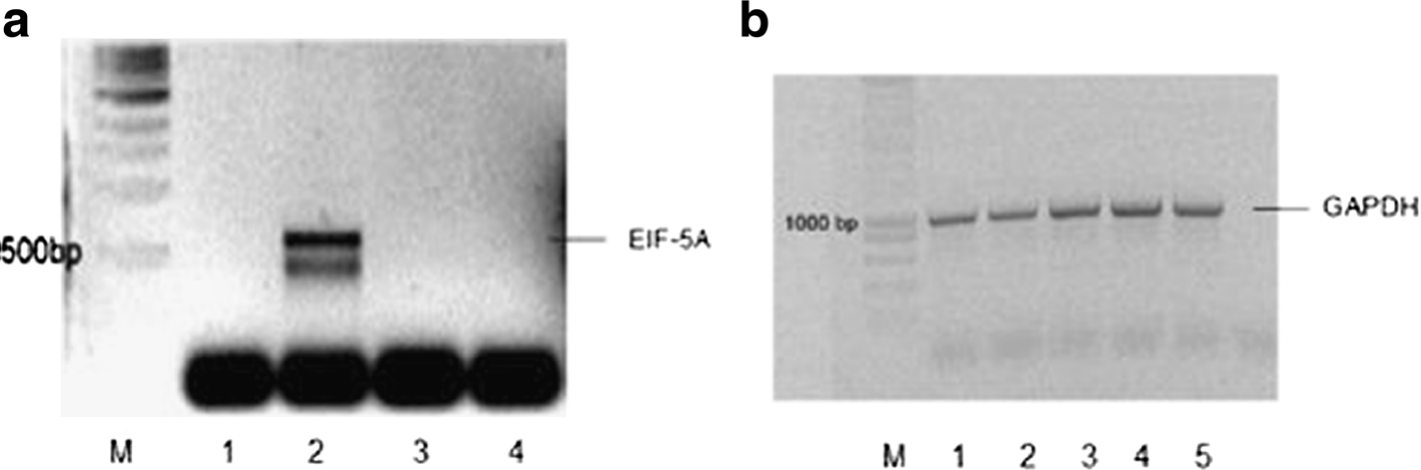
**A) Silencing of parasitic EIF-5A by RNAi in 293 T cells and subsequent monitoring by RT-PCR.** A cotransfection was performed with: 1) no transfected DNA; 2) recombinant, plasmodial *eIF-5A* expression plasmid with the 483 bp cDNA; 3) EIF-5A-shRNA construct P#18; 4) aquaporin-5-specific siRNA. **B**) The 983 bp GAPDH amplificate was used as an internal control in the transfected mammalian cell line.

Two different DHS short hairpin RNAs (shRNAs), #43 and #176 (see Materials and Methods section), expressed from the *pSilencer1.0-U6* vector were applied to knock down the DHS protein from *P. falciparum.* The shRNA #43 targets the *dhs* sequence at nucleotide positions 337–358, while shRNA #176 targets the *dhs* sequence at nucleotide positions 1269–1290 within the *P. falciparum* mRNA. Both constructs were individually cotransfected with plasmodial DHS expression vector into 293T cells to verify the expected degradation of the *dhs* transcript. The results obtained by RT-PCR analysis show a significant knock-down of plasmodial *dhs* transcript by the shRNA P #176 construct (Figure 1A, lane 4), as opposed to when the shRNA P #43 was expressed (lane 5). By contrast, a control siRNA which lacks complementary sequences in the human genome did not negatively affect the abundance of the *Plasmodium* transcript with the expected size of 612 bp (amino acid positions 208–412) (lane 1). To exclude any off-target effects a number of control experiments was performed. As a positive control the recombinant plasmodial DHS expression vector was transfected alone into 293T cells. Following RT-PCR the cDNA fragment of 612 bp was detected (lane 3). No transcript could be observed when untransfected 293Tcells were analyzed (lane 2). Next, we amplified the human GAPDH sequence, representing a housekeeping gene, to control the various cotransfections. As shown, the presence of the expected GAPDH amplificate was detected in all analyzed samples (Figure 1B), suggesting that the silencing effect of the DHS siRNA used is specific since the *dhs* amplificate does not show any homology to its human orthologue. In a separate set of experiments we applied 4 different shRNAs to knock down the eIF-5A precursor protein. The *pSilencer1.0-U6* vectors expressing different eIF-5A shRNAs (#5, #6, #7, and #18; see Materials and Methods and (Additional file 1: Figure S 1) were individually cotransfected with plasmodial eIF-5A expression vector into 293T cells. Again, the monitoring of eIF-5A transcript abundance was performed by RT-PCR. From the 4 tested eIF-5A siRNAs only shRNA #18 (Figure 2A, lane 3) was capable of completely downregulating the plasmodial eIF-5A mRNA level in 293T cells. For all other constructs an *in vitro* knockdown was unsuccessful (our own data; not shown) *.*

Control reactions with non-transfected cells (Figure 2A, lane 1) and eIF-5A shRNA #18 cotransfected with the aquaporin-specific siRNA (Figure 2A, lane 4) did not change the silencing effect. Although eIF-5A is a highly conserved protein in eukaryotes its nucleic acid sequence is significantly divergent in comparison to its human orthologue and thus amplificates from endogenous eIF-5A are not expected. Again, we monitored the presence of GAPDH by RT-PCR in all transfections (Figure 2B) independently of the presence of the siRNA construct.

To further validate the RT-PCR experiments the limit of detection for the corresponding mRNAs i.e. *eIF-5A* and *dhs* was determined. Titrated *dhs*-specific mRNA resulted in a limit of detection of 20 ng while *eIF*-5A-specific mRNA could only be detected at a concentration of 200 ng. Optimal primer binding was determined for eIF-5A-specific primers at a cDNA concentration of 130 ng and for *dhs-*specific primers at a cDNA concentration of 650 ng (data not shown).

In sum, these data demonstrated that *Plasmodium-*specific eIF-5A and DHS sequences can in principal be silenced by RNAi.

### Monitoring *in vivo* silencing of eIF-5A and DHS in erythrocytic stages after infection of NMRI mice with transgenic schizonts from P. berghei

With regard to the *in vitro* results, we investigated the silencing effect of the expressed DHS-specific and eIF-5A specific shRNAs in an *in vivo* rodent model of *P. berghei* ANKA strain [24]. Infection of NMRI mice with P. *berghei* ANKA wild type strain leads to experimental cerebral malaria within 6 to 10 days p. i. although the parasitemia is only in the range of 3–5% infected erythrocytes. In case of the infectious but non lethal phenotype *P. berghei* strain NK56, the infected mice succumb to high parasitemia within 80 days p.i. without cerebral malaria.

**Figure 3:**
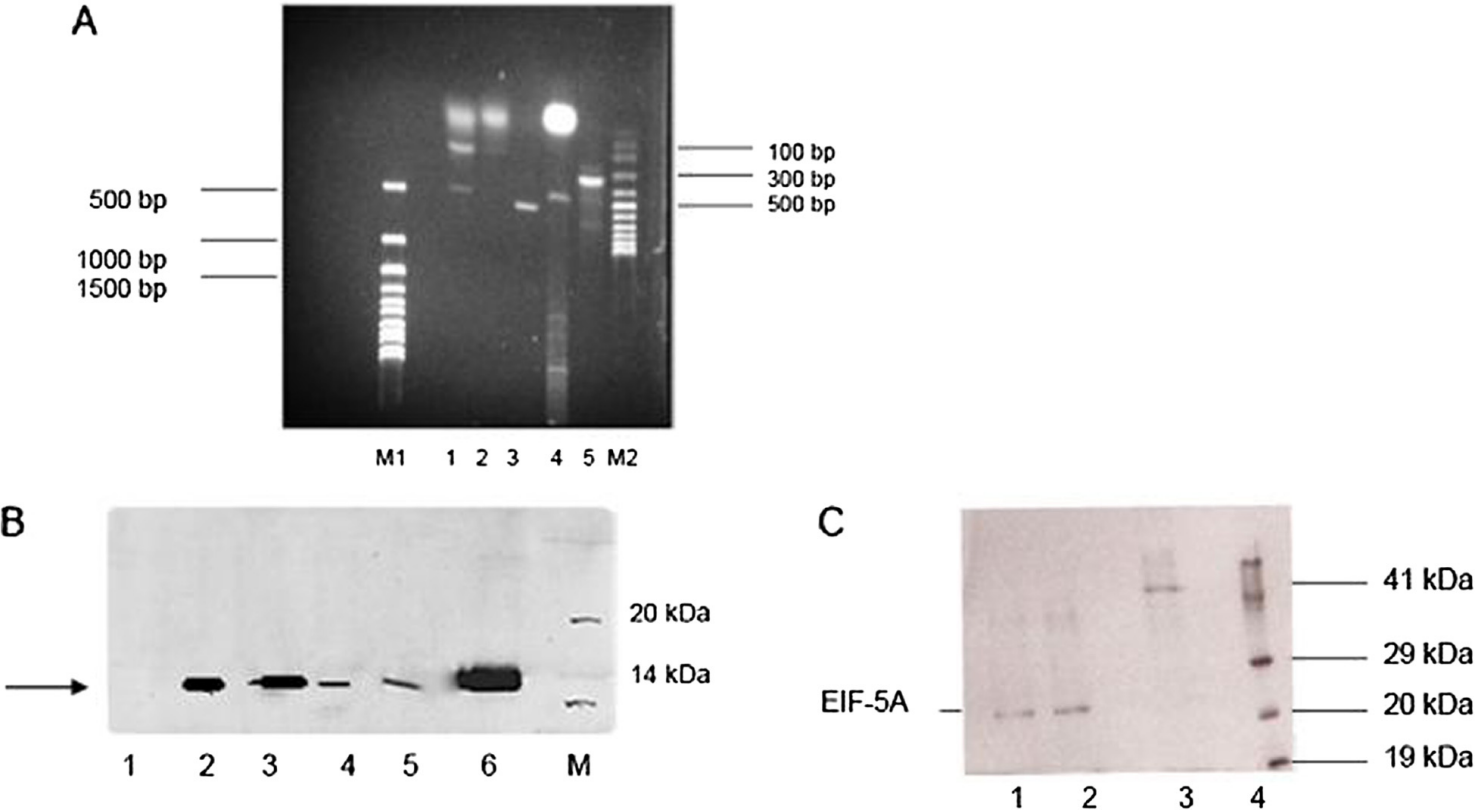
**A) Monitoring in vivo silencing of parasitic eIF-5A by RT-PCR in RBCs of infected NMRI mice 2 days post infection.** NMRI mice were infected with transgenic schizonts harbouring the expressed shRNA P#18. M1) 1 kb ladder (LifeTechnologies, Karlsruhe, Germany); 1) non-transfected 293T cells 2) EIF-5A-siRNA; 3) A positive control for the quality of cellular RNA is the 548 bp amplificate generated with α-tubulin gene-specific primers from *P. berghei*; 4) A PCR-control reaction with *eIF-5A*-gene specific primers from *P.vivax* generates a 448 bp cDNA fragment; 5) Amplificate of 323 bp obtained in a RT-PCR control reaction from a 1.4 kb kanamycin mRNA; M2) 100 bp ladder (Life Technologies, Karlsruhe, Germany). **B**) Western Blot analysis of parasitic sh-eIF-5A expression from transgenic schizonts after infection of NMRI mice. Protein extracts were generated from: 1) shEIF-5A RNA #P18; 2) shDHS-RNA; #P176; 3) protein extract from RBCs infected with *P. berghei* ANKA strain and 4) mock strain (without transfected shRNA); 5 and 6) different protein concentrations of the EIF-5A histidine-tagged, purified protein. 7) Standard protein marker (Roth). Polyclonal-antibody against EIF-5A protein from *P. vivax* was applied in a concentration of 1:1000 which detected the protein band with a molecular weight of 20 kDa. The protein concentrations of the extracts were 10 μg/μl. **C**) Confirmation of the specificity of the used anti-eIF-5A antibody by Western Blot analysis. 1) Protein extract prepared from NMRI infectected mice expressing the #18 eIF-5A-specific shRNA, supplemented with recombinant eIF-5A protein from *P.vivax*; 2) purified, recombinant EIF-5A protein from *P.vivax*; 3) Protein extract prepared from NMRI infected mice expressing the #18 eIF-5A-specific shRNA. The protein concentration was 10 μg in each lane.

In a first step DHS-specific shRNA #176 or eIF-5A-specific shRNA #18 expressed from pSilencer 1.0-U6 vector was transfected into schizonts, the late developmental stage of the parasite. These transgenic schizonts were applied to NMRI mice for infection. *In vivo* gene silencing was monitored in the animals’ erythrocytes at day 2 post infection by RT-PCR as before. Infection with schizonts containing the eIF-5A-specific shRNA #18 vector (Figure 3A lane 2) led to a complete disappearance of the respective transcripts, at least within the detection level of this assay. By contrast, the eIF-5A sequences were clearly detected in the erythrocytic stage after infection with schizonts, which were transfected with the *dhs*-specific shRNA #176 vector (Figure 3A, lane 1). Several control reactions were applied. The RT-PCR reactions of a kanamycin control RNA of 1.2 kb (Figure 3A, lane 5) and that of the recombinant eIF-5A plasmid from *P. vivax* was monitored, resulting in amplification products of approximately 323 bp and 448 bp, respectively (Figure 3A, lanes 5 and 4). In parallel we confirmed the quality of the total cellular RNA preparation for the presence of the α-tubulin II sequences, which are expressed in the asexual blood stages of *Plasmodium* (lane 4).

Next we monitored the effect of *in vivo* eIF-5A silencing on the protein level. As shown in Figure 3B, eIF-5A protein was absent in NMRI infected mice with transgenic schizonts expressing the #18 eIF-5A-specific shRNA. In these experiments a polyclonal anti-eIF-5A antibody raised against the highly conserved *P. vivax* protein (96% identity) was used. NMRI mice infected with transgenic schizonts expressing the #176 DHS-specific shRNA construct showed that the unmodified or hypusinated eIF-5A protein was present (Figure 3B, lane 2). This result implies that the #176 DHS-specific shRNA construct exclusively affects the DHS protein. Although eIF-5A is not modified and is mostly abundant in its unhypusinated form, it is recognized by the polyclonal anti-eIF-5A antibody (Figure 3B, lane 2). The results further support the observation that the RNA encoding the *eIF-5A* gene is present in the erythrocytic stages after infection with schizonts expressing the DHS *-*shRNA #176 (Figure 3A, lane 4). The polyclonal antibody detected the eIF-5A protein with a size of 17,75 kDa in the *P. berghei* ANKA strain (Figure 3B, lane 3) as well as in the mock control strain (Figure 3B, lane 4), while the eIF-5A protein from *P. vivax* displayed the expected molecular mass of approximately 20 kDa (lanes 5 and 6).

To further support the specificity of the polyclonal anti-EIF-5A antibody, protein extracts obtained from the infected NMRI mice expressing the #18 eIF-5A-specific shRNA were spiked with purified eIF-5A protein from *P. vivax* (Figure 3C, lane 1). The *P. vivax* anti-eIF5A antibody clearly detected the EIF-5A protein in the respective extract (lane 1) while EIF-5A protein was absent in the crude extract of *P. berghei* ANKA strain (Figure 3C, lane 3). Moreover, the purified, recombinant eIF-5A protein was clearly recognized by the antibody (Figure 3C, lane 2). These data suggest that an *in vivo* knockdown of eIF-5A is possible.

**Figure 4:**
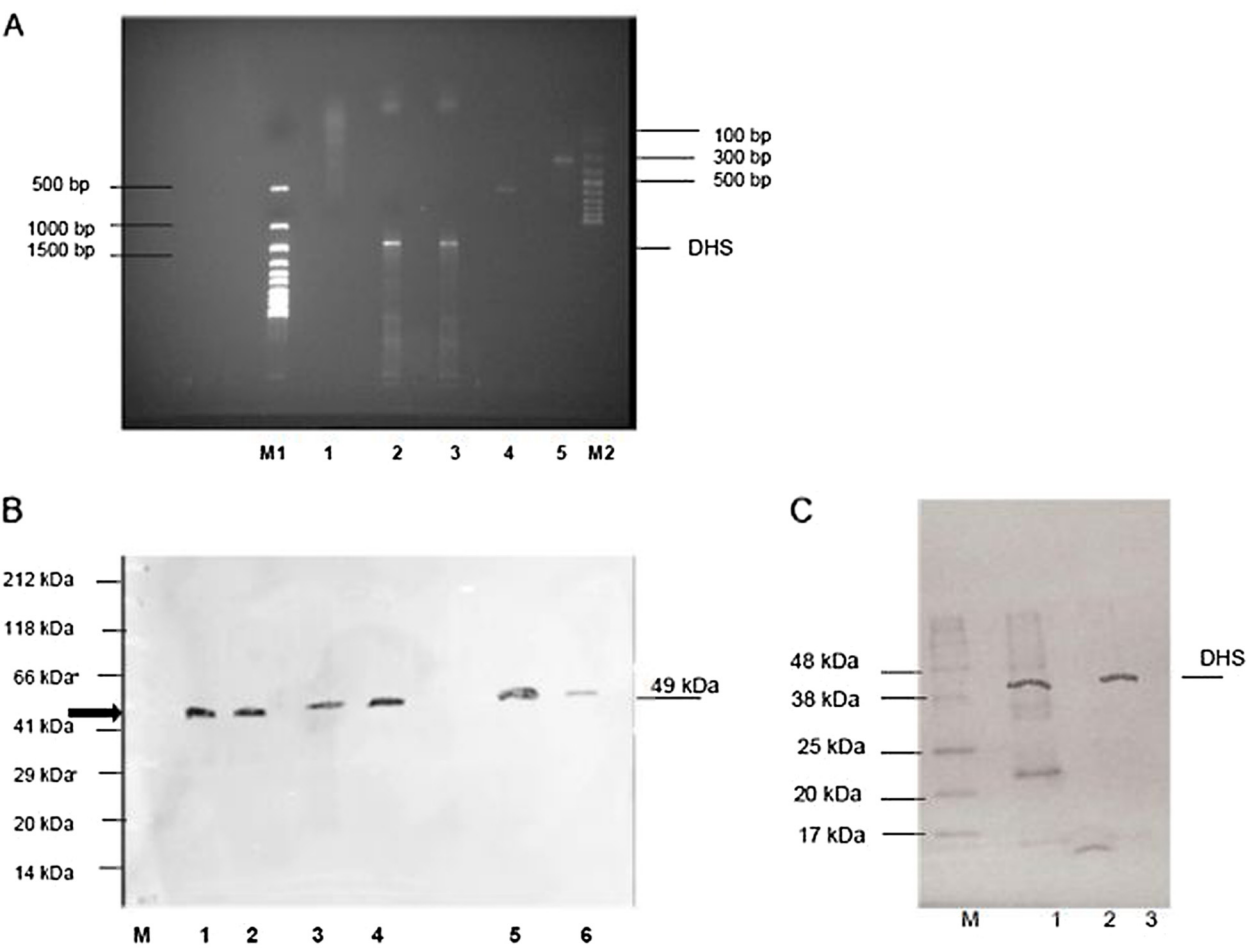
**A) Monitoring the in vivo knockdown of*P. berghei*infected schizonts transgenic for the expressed plasmodial DHS shRNA by RT-PCR two days post infection with NMRI mice.** NMRI mice were infected with transgenic schizonts transfected with the plasmodial shRNA P#176 construct. M1) 1 kb ladder (LifeTechnologies, Karlsruhe, Germany); 1) DHS-shRNA; 2) EIF-5A-shRNA; 3) Amplification of the recombinant pcDNA3 vector carrying the *dhs* gene from *P. falciparum* generates a cDNA fragment of 1491 bp. 4) Quality control of total, cellular RNA by amplification of a 548 bp fragment with α-tubulin gene-specific primers from *P. berghei*; 5) PCR-control of recombinant eIF-5A (448 bp) expression vector with eIF-5A primers; M2) 100 bp ladder (LifeTechnologies, Karlsruhe, Germany) **B)***In vivo* silencing of plasmodial DHS monitored by Western blot analysis after infection of NMRI mice with transgenic schizonts expressing shDHS. 1 and 2) Two different concentrations of purified, human DHS protein; 3) PB ANKA wild type strain protein extract 4) Mock strain protein extract; 5) eIF-5A shRNA P#18; 6) DHS- shRNA P#176. **C**) Validation of anti-DHS antibody specificity in crude protein extracts prepared after infection of NMRI mice expressing plasmodial DHS #176 shRNA construct. M) Blueeye Marker; 1) crude protein extract from infected NMRI mice with plasmodial DHS #176 shRNA construct and supplemented with recombinant human protein; 2) crude protein extract from infected NMRI mice with plasmodial DHS #176 shRNA construct; 3) purified recombinant human DHS protein. The protein concentration was 10 μg in each lane.

A DHS-specific RT-PCR was performed to control formation of the 1248 bp cDNA fragment in the erythrocytic stages after infection of NMRI mice with transgenic schizonts harbouring the DHS-shRNA #176 and the eIF-5A #18 construct (Figure 4A, lanes 1–2). A *dhs*-specific transcript was not detectable in the #176-infected (shRNA expressing) erythrocytes (lane 1), while it was present in the #18-infected (shRNA expressing) erythrocytes (lane 2) and in the control reaction with plasmodial *dhs*-specific primers (lane 3). Additionally, the quality of the cellular RNA was confirmed with *P. berghei* specific α-tubulin primers (lane 4) by reverse transcription using a 1.2 kb Kanamycin-mRNA (lane 5). In parallel we controlled *in vivo* silencing of DHS levels by Western blot analysis (Figure 4B). A polyclonal anti-DHS antibody raised against the human DHS protein detected the predicted size of 41 kDa when different concentrations of purified human DHS were applied (lanes 1 and 2). Results from an amino acid alignment showed that human DHS isoform1 shares 57% amino acid identity to the *P. falciparum* 3D 7 orthologue, 58% amino acid identity to the *P. vivax* orthologue and 56% identity to *P. berghei.* These highly conserved amino acid regions were apparently recognized by the human antibody. Protein extracts prepared after infection with *P. berghei* (lane 3) and mock strain (lane 4) showed the expected 49 kDa orthologue of DHS. DHS was completely abundant in the eIF-5A-shRNA mutant #18 (lane 5) and a faint band was visible in the DHS-shRNA mutant (lane 6), although no cDNA could be detected in a RT-PCR reaction.

Again, as already performed with eIF-5A, the specificity of the human anti-DHS antibody was confirmed. Protein extracts prepared from the infected NMRI mice harbouring the expressed sh-RNA construct #176 were supplemented with recombinant, human DHS protein (Figure 4C, lane 1). The human anti-DHS antibody clearly detected the recombinant human protein (lane 3) and the added DHS protein (lane 1). However, in the extract with the plasmodial shRNA #176 a DHS signal was absent (lane 2). These data demonstrate the validity of this antibody.

### Monitoring parasitemia after infection of schizonts transfected with eIF-5A- and DHS-specific siRNA

With respect to the *in vitro* silencing data, *P. berghei* purified schizonts were transfected with either the eIF-5A shRNA construct (P #18) or the DHS shRNA (P #176) construct. In both cases, transfected cells were tracked for infection in recipient outbred NMRI mice without any selection pressure.

**Figure 5:**
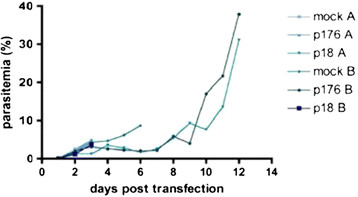
**Parasitemia of outbred infected recipient mice post transfection with schizonts transgenic for parasitic eIF5A-shRNA or DHS-shRNA.** Infection with each construct was performed in two different independent experiments with two mice per condition. Pale blue triangles and blue points represent the curves for the determined parasitemias post infection with the shDHS P#176 in two mice. Pale blue upside down triangles and blue squares represent the monitored parasitemia with the expressed eIF5A-sh P#18. The parasitemia for the mock control strain is represented by the pale blue dot and the pale blue rhomb.

In two independent, different sets of experiments infection of mice was monitored after transfection of recombinant schizonts expressing either the P #176 DHS-shRNA, or the P #18 construct (eIF-5A-shRNA) (Figure 5). As a control, an infection was performed using a mock strain, which was not transfected with DNA. From day 2 to day 10 post infection, parasitemia was significantly lower in both lines compared to the untransformed mock strain. By contrast, the mock strain displayed a parasitemia of 9% at day 6 post infection, compared to the transfected parasites with the DHS-shRNA (4.5%) or the eIF-5A-shRNA. After 9 days post infection, parasitemia increased significantly in both infection experiments, harbouring either the transgenic schizonts with the DHS-shRNA or the eIF-5A-shRNA.

### Studies on the effect of cytokines on iNos production due to posttranslational modification of eIF-5A

**Figure 6:**
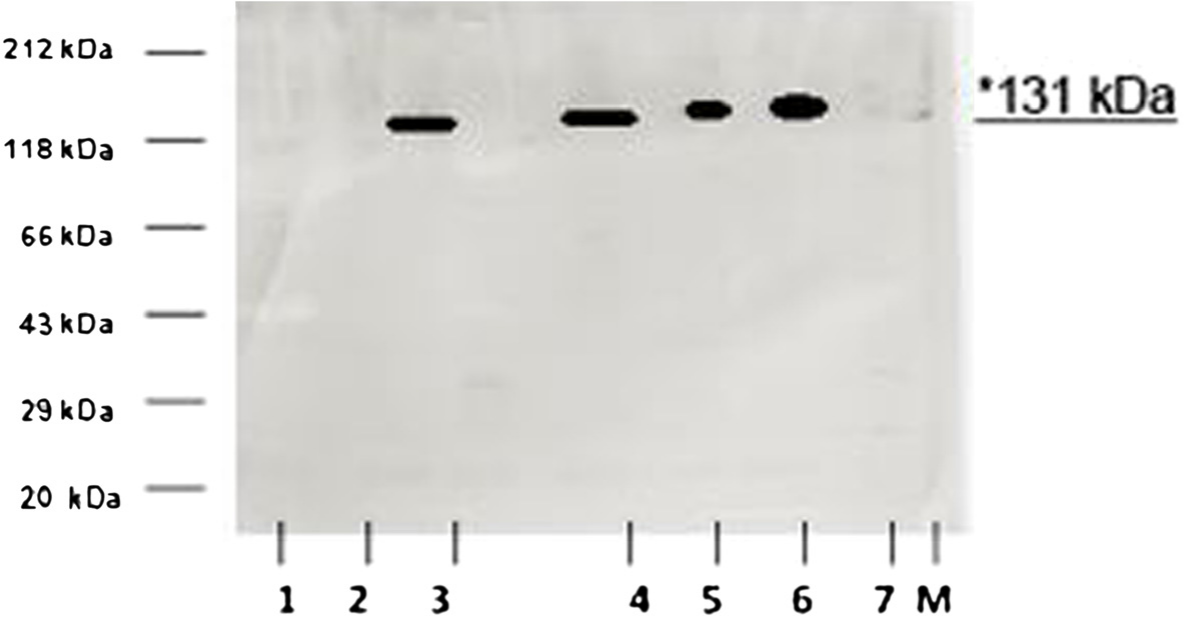
**Cytokine signaling for human iNos2 translation is dependent on the hypusine pathway during the infection of*Plasmodium.*** Western Blot analysis was performed with equal amounts of protein (10 μg) extracted from the infected erythrocytic stages with transgenic schizonts from *P. berghei* ANKA strain 1) protein extract prepared from serum after infection with schizonts harbouring the expressed plasmodial DHS-shRNA or 2) the eIF-5A-siRNA expression construct; 3) *P. berghei* ANKA strain; 4) induced and 5) non- induced Jurkat cells; 6) Mono Mac 1 cells; 7) HeLa cells; M) Standard protein marker Roth, St. Leon, Germany. Detection of the iNos2 protein with a molecular size of 131 kDa was performed with a human anti-Nos2 antibody in a dilution of 1:1000.

To investigate a possible link between cytokine signaling and translation of *iNos2* mRNA of the host, Western blots were performed with protein extracts from serum of the infected erythrocytic stages of NMRI mice. Figure 6 shows that signals for iNos2 were absent in serum p. i. after DHS silencing with construct P #176 and eIF-5A-shRNA construct P #18 (Figure 6, lanes 1 and 2), while iNos2 protein with a molecular size of approximately 131 kDa was detectable in the *P. berghei* ANKA strain infected erythrocytes (Figure 6, lane 3). Most notably, prominent signals for iNos2 protein were detected in immortalized T cells (Jurkat cells) (Figure 6, lane 4 uninduced and lane 5 induced) and a monocytic cell line (Mono Mac) (Figure 6, lane 6). No signal was obtained in HeLa cells (lane 7).

There was no difference in signal intensity between induced and uninduced cells probably due to the induction by ionomycin/PMA (phorbol 12-myristate 13-acetate), which might not be the correct inductor to stimulate cytokine cell signaling.

To further support these results nitric oxide was quantified in a colorimetric assay after an enzymatic conversion of nitrate to nitrite by the enzyme nitrate reductase followed by detection of nitrite as a colored azo dye product. The amount of the formed nitrite and nitrate from nitric oxide was approximately 20-fold lower in the serum after infection of mice with the shRNA construct P #18 (108,8 μM/L) (Table 1) and 18-fold lower with the shRNA construct P #176 (120 μM/L) (Table 1) in comparison to the wild type (2260,5 μM/L).

**Table 1:** **Colorimetric determination of nitric oxide formation as nitrate and nitrite in sera from infected mice obtained after*P.berghei*ANKA strain infection and after infection with schizonts harbouring the expressed plasmodial DHS shRNA #176 or plasmodial EIF-5A shRNA #18**

**Nitrate and nitrite [μmol/L]**	**Wild type and transfectants**
2200,5	*P. berghei* ANKA wild type
120	DHS-specific shRNA # 176
109	EIF-5A-specific shRNA # 18

Nitrate and nitrite determination after infection of mice with transgenic schizonts expressing plasmodial DHS and EIF-5A shRNAs.

## Discussion

Hitherto, the biological function of the unusual amino acid hypusine has not been studied in *Plasmodium*. Previous studies showed that hypusination of eIF-5A is important for cell proliferation of the parasite [11]. Deoxyhypusine synthase (DHS), which catalyzes the first step in the hypusine pathway has been recently targeted by a small molecule inhibitor, CNI-1493, which blocked parasitic growth *in vitro* and *in vivo*[22]. Another excellent way to study the biological function of this posttranslational modification in more detail is a genetic analysis by loss of function of the proteins involved in hypusine biosynthesis.

For the future it will be an important issue to pursue a targeted, stable gene disruption of the *dhs* and *eIF-5A*genes in *Plasmodium,* since their exact function in the erythrocytic life cycle stages is still unknown. To date gene disruption by insertion strategy has been successfully shown in the rodent model of *P. berghei* and it is partly working in the intraerythrocytic schizogeny *of P. falciparum*[24,25].

The understanding of cerebral malaria (CM) pathogenesis is still rudimentary [26]. Our results clearly demonstrate that the hypusine pathway in *Plasmodium* supports at least two different hypotheses in the pathogenesis of cerebral malaria i.e. the sequestration theory and the inflammation hypothesis. One of the underlying mechanisms of cerebral malaria pathogenesis is the adherence of parasitized red blood cells to vascular endothelial cells by parasite specific proteins. Infected NMRI mice transfected with schizonts transgenic for plasmodial eIF-5A- or DHS-specific shRNA showed a 50% reduced parasitemia in comparison to the untransfected control within 2 to 9 days post infection. This may indicate the preventing of parasitic sequestration.

In a first approach to test the possibility whether a knockdown of DHS and its precursor protein eIF-5A is possible in *Plasmodium*, an in vitro knockdown by RNAi was performed since an unequivocal demonstration that the *Plasmodium* genome contains any of the conserved RNAi machinery genes or enzymes is to date missing. In the past, RNAi in circulating malaria parasites was performed showing 50% reduction at the expression level of berghepains which are homologues of cysteine proteases in *Plasmodium*[27]. For the siRNA experiments, a strategy to reduce gene expression in cultured cell lines with *pSilencer1.0-U6* vectors producing the respective shRNAs from the U6 promotor was selected.

The data indicate that an *in vitro* knockdown of eIF-5A with four different shRNAs was not completely ablating eIF-5A expression except for the shRNA # P18 in 293 T cells (Figure 2A, lane 3) which markedly reduced the eIF-5A transcript level. These four shRNA constructs of eIF-5A were targeted all over the *eIF-5A* sequence. The eIF-5AshRNA #18, which targets positions 163–184 in the *eIF-5A* nucleic acid sequence, caused a complete decrease in *eIF-5A* mRNA levels. These results are in agreement with the structural model of human eIF-5A1 [30], which consists of two domains, a basic N-terminal domain with the hypusine loop and an acidic -terminal domain connected by a hinge. Within the basic N-terminus, the hypusine modification covers amino acid positions 46–54 i. e. nucleic acid positions 138–162 which are very close to the 3’ prime end of the hypusine loop.

By contrast eIF-5A shRNA #7 targets position 115–136, which is proximal to the 5’-end of the loop, does not affect mRNA abundance. It is likely that the secondary structure of the hypusine loop at this position might block the degradation of the specific mRNA [28]. Taken together, from four tested shRNAs, only one, the eIF-5A-specific shRNA #18 caused a considerable decrease of the *eIF-5A* transcript *in vitro*.

Two DHS-shRNAs, #43 and #176, targeting nucleotide positions from 337–358 bp and 1269–1290 bp, respectively, were employed for an *in vitro* knockdown of DHS from *Plasmodium*. Surprisingly, the DHS-shRNA construct #176 was successful to downregulate the *dhs* transcript significantly (Figure 1A, lane 5), although the targeted sequence did not cover the active site of the enzyme within the amino acid region between Lys^287^ and Glu^323^[28,29].

Subsequently, monitoring of *in vivo* silenced *P. berghei* blood stage parasites transgenic for either eIF-5A-shRNA or DHS-shRNA post transfection was performed by RT-PCR. In case of the eIF-5A-shRNA containing blood stages the *eIF-5A* transcript was not present (Figure 3, lane 2), while in erythrocytes with the DHS-shRNA (Figure 3A, lane 2) the *dhs* cDNA was not abundant (Figure 4A, lane 1). However, the *eIF-5A* transcript was detectable, suggesting that the silencing effect is rather specific.

Moreover, these results were confirmed by Western blot analysis where the 17,75 kDa eIF-5A protein was absent in the transgenic *P. berghei* ANKA parasites harbouring the eIF-5A-specific siRNA. Both proteins, i.e. the *P. falciparum* and the *P. berghei* homolog share amino acid identities of 73%. In a control experiment the antibody raised against the eIF-5A protein from *P. vivax* crossreacted with the eIF-5A homologue from the mock strain and the *P. berghei* ANKA strain resulting in a protein of 17,75 kDa [30] (Figure 3B, lanes 3 and 4). To monitor suppressed DHS expression a polyclonal human antibody was applied which detected the *P. berghei* orthologue of 49 kDa (Figure 4B, lanes 3 and 4) in the mock control and the *P. berghei* ANKA strain. By contrast a faint band was detected in the DHS siRNA mutant suggesting that the gene may not be silenced completely.

The inflammation hypothesis in cerebral malaria implies that brain damage is a result of the inflammatory response of the human host to the parasite in the central nervous system (CNS). The production of proinflammatory cytokines like IL-1β, TNF-α, IFN-γ leads to secretion of nitric oxide which kills the parasite. It has been recently reported that hypusinated eIF-5A is required in part for the nuclear export and translation of iNos-encoding mRNAs in pancreatic, stressed ß-cells after release of proinflammatory cytokines [17]. To test this hypothesis the host iNos2 protein levels were monitored in serum after infection with *P. berghei ANKA* strain and the two transfected shRNAs P #176 (DHS) and P #18 (EIF5A), since a parasitic nitric oxide synthase is absent. We could clearly demonstrate that in both mutants there is no response to cellular stress i.e. induction of inducible nitric oxide synthase (iNos2) of the human host once modification of eIF-5A is interrupted by silencing of either parasitic DHS or eIF-5A. However, nitric oxide synthase is induced 20-fold after infection with the wild type *P. berghei* ANKA strain in comparison to the shRNA mutants P #176 (DHS) and P #18 (EIF5A) with a 18-fold and 20-fold lowered formation of nitric oxide. These findings do not only prove a link between the hypusine pathway and iNos production but also broaden our understanding of the CM malaria pathology and implicate alternative strategies for therapy. Similar results have been obtained in DHS heterozygous knockout mice with attenuated cytokine signalling as evidenced by reduced nitric oxide synthase production [31].

Malaria patients often present with hypoargininemia [32], and metabolomic studies of *Plasmodium falciparum* during its 48 h intraerythrocytic life cycle reveal nearly complete depletion of L-arginine levels. Nitric oxide synthase is induced by arginine and catalyzes the reaction to nitric oxide (NO) and urea. However, in cerebral malaria there is a lack of nitric oxide due to the presence of parasite-specific arginase which leads to a depletion of arginine and subsequent downregulation of host-specific nitric oxide synthase. This may allow the parasite to evade a NO-dependent immune response in the host since NO is deleterious to parasite proliferation [33]. During *Plasmodium berghei* ANKA infection in mice exogenous nitric oxide decreases brain vascular inflammation, leakage and venular resistance [17,18] and protects against cerebral malaria.

Finally, the crystal structure of *Plasmodium* arginase has been resolved recently and indicates a low complexity region [33] which is largely disordered and its deletion does not significantly compromise enzyme activity. Moreover, disruption of *P. falciparum* arginase led to an apparent reduction in liver stage infection.

## Conclusions

Although it has been previously suggested that RNAi is not functional in *Plasmodium*, a putative, non-canonical RNAi pathway might exist in malaria parasites. *In vivo* knockdown of eIF-5A and DHS by expression of shRNAs after infection in a rodent model decreased parasitemia intermittently in the development of cerebral malaria. The data are similar to the related but non-lethal phenotype *P. berghei* ANKA NK 65. These results might be of further interest to study the function of hypusine modification with respect to malaria infection and therapy.

## Materials and methods

### Ethics statement

All animal experiments were performed under FELASA category B and GV-SOLAS standards. Animal experiments were approved by German authorities (Regierungspräsidium Karlsruhe, Germany).

### siRNAs (small interfering RNAs) for targeting DHS and eIF-5A

The following double stranded siRNAs were used and synthesized by Ambion, Karlsruhe, Germany: RNAi for DHS and EIF5A knockdowns, siRNA duplexes were used which were obtained by annealing the RNA oligonucleotides synthesized by Ambion, Karlsruhe, Germany. The oligonucleotides (3000 mM for *dhs*, 1500 mM for eIF-5A) were phosphorylated in a reaction volume of 20 μl with 3 Units polynucleotide kinase (10 U/μl) (Roche Diagnostics, Penzberg, Gemany) at 37°C for 45 min. The reaction was stopped on ice for 1 min. An annealing reaction was performed at 95°C with subsequent cooling of the reaction to room temperature overnight.

After annealing the siRNA duplexes were cloned into p*Silencer* 1.0-U6 vector before transfection into 293T cells or schizonts.

For the DHS knockdown #43, RNA oligonucleotides 5’- UGUUAGUGAAGAUCUUAAUtt-3’ and 5’-AUUAAGAUCUUCACUAACAtt-3’ were applied targeting the nucleotide positions 337–358 in the plasmodial *dhs* cDNA. For the DHS knockdown #176, RNA oligonucleotides 5’- UGAGGAAUGGUGCUGAUUUtt-3’ and 5’-AAAUCAGCACCAUUCCUCAtt-3’ were applied which targeted nucleotide positions 1269–1290 in the *dhs* cDNA.

For the eIF-5A knockdowns 4 different siRNA duplexes were generated. For the EIF-5A knockdown #5, RNA oligonucleotides 5’- ACGGCCACGUGAUGCUAAAtt-3’ and 5’- UUUAGCAUCACGUGGCCGUtt-3’ were applied targeting nucleotide positions 81–102 in the *P. vivax* eIF-5A cDNA. For the EIF-5A knockdown #6, RNA oligonucleotides 5’- AGGAGCAUCCUUGCAAAGUtt-3’ and 5’- ACUUUGCAAGGAUGCUCCUtt-3’ were applied which targeted nucleotide positions 99–120; for EIF-5A knockdown #7, RNA oligonucleotides 5’-AGUGGUAGAUUACUCCACGtt-3’ and 5’- CGUGGAGUAAUCUACCACUtt-3’ were used for targeting nucleotide positions 115–136. For eIF-5A knockdown #18, RNA oligonucleotides 5’- CUGAGUUGCAGCUGAUUGAtt-3’ and 3’- UCAAUCAGCUGCAACUCAGtt-5’ were applied which targeted the *eIF-5A* gene at nucleotide positions 163–184.

### Construction of pSilencer1.0-U6 vector with double stranded siRNA of DHS and eIF-5A

20 μg of (Ambion/Invitrogen, Karlsruhe, Germany) was double digested with *Eco*RI/ *Apa*I (20 U) in a reaction volume of 20 μl and dephosphorylated with calf intestine alkaline phosphatase (CIP) (MBI Fermentas, St. Leon Rot, Germany) (1 U/μl) for 1 hour at 37°C. The double digested vector was gel-purified according to the Mini Elute Gel Extraction Kit protocol from Qiagen, (Hilden,Germany). Ligation of the annealed oligos was performed with the ligation kit from Roche Diagnostics, (Penzberg, Germany). Positive constructs were analysed after double digestion with *Apa*I and *Hind*III.

### Cloning the full length dhs cDNA and eIF-5A cDNA into eukaryotic pcDNA3 vector

Amplification of the *dhs* gene was performed from the recombinant pet- *Blue1* plasmid (Novagen, Darmstadt,Germany) from *Plasmodium falciparum* with primers containing recognition sites for *Eco*RI (restriction site is underlined) *dhs *forward 5’-TTT GAATTCATGGTGGATCACGTTTC-’3’ and *Not*I *dhs* reverse 5’- TTT GCGGCCGCTCACATATCTTTTTTCCTC- 3’. The resulting fragment of 1491 bp was digested with *Eco*RI and *Not*I and ligated into *Eco*RI/ *Not*I treated pc *DNA3* vector (Invitrogen, Darmstadt, Germany) and re-sequenced. For construction of an *eIF-5A* cDNA containing pcDNA3 vector, the *eIF-5A* nucleic acid sequence was amplified from a recombinant plasmid pSTBlue-1 *Acceptor*™ vector (Novagen, Darmstadt, Germany) with primers containing *Eco*RI *eIF-5A*forward 5’ -AAA GAA TTC ATG TCA GAC CAC GAA AC-3’ and *Not*I *eIF-5A*reverse 5’-TTT GCG GCC GCC TAG GAG GAC AAC TCC-3’ restriction sites.

### Cotransfection of pSilencer1.0-U6 vectors into 293 T cells

In a 6 well microtiter plate 7x10^5^ 293T cells were seeded in all 6 wells. Four different sets of cotransfections were performed: DHS; i) *P. falciparum dhs* cDNA in pcDNA3 (0.3 μg), ii) *P. falciparum dhs* cDNA in pcDNA3 and premade scramble II duplex negative control siRNA (1.0 μg), iii) *P. falciparum dhs* cDNA in pc *DNA3* and DHS- specific shRNA construct #43 (1.0 μg), iv) *P. falciparum dhs* cDNA in pcDNA3 and DHS-specific shRNA construct #176 (1.0 μg). The various transfections were mixed with transfection mix (total vol. 400 μl), which contained Opti-MEM® (Invitrogen, Karlsruhe, Germany) and polyethylenimine (PEI) (4 μl/μg), and were added to the cultures. After 10 min of incubation at room temperature, the culture supernatants were substituted by 2 ml of DMEM (Dulbecco's Modified Eagle's Medium) (Invitrogen, Karlsruhe, Germany) and the cell cultures were incubated overnight at 37°C. The next day, medium was changed and supplemented with streptomycin (60 μg/ml). Prior to transfection, the cells were washed with PBS-buffer (phosphate buffer saline).

Cotransfection of *P. falciparum* eIF-5A pcDNA3-based expression vector in combination with 4 different sets of siRNA vectors was performed according to a protocol from Invitrogen (Karlsruhe, Germany): i) *P. falciparum* eIF-5A expression vector (0.3 μg) and aquaporin-5 specific-siRNA (2.7 μg) ii) *P. falciparum* eIF-5A expression vector (0.3 μg) and eIF-5A-specific shRNA construct #18 (2.7 μg), iii) *P. falciparum* eIF5A expression vector (0.3 μg) and eIF-5A-specific shRNA #6 (2.7 μg), iv) *P. falciparum* eIF-5A expression vector (0.3 μg) and eIF-5A shRNA construct #7 (2.7 μg), v) *P. falciparum* eIF-5A expression vector (0.3 μg) and eIF-5A shRNA #5 (2.7 μg).

### Isolation of cellular RNA

Isolation of total cellular RNA was performed according to the RNeasy Plant Mini Kit (Qiagen, Hilden, Germany). The quality of the isolated RNA was verfied by agarose gel electrophoresis and the quantity and purity was determined by UV spectrometry.

### RT-PCR analysis of eIF-5A and DHS silencing *in vitro* and *in vivo*

To monitor the silencing of eIF-5A and DHS, RT-PCR was performed according to a protocol from the AccessQuick™ RT-PCR System (Promega, Mannheim, Germany). For the RT-PCR reaction gene specific primers for *eIF-5A*forward 5’-ATGTCAGACCACGAAACGT-3’/eIF-5A reverse 5’-CTAGGAGGACAACTCCTTCACCGC- 3’ and *dhs forward* 5’-ATAGTGCCTAATGATAATTA -3’/dhs reverse 5’-AACCTCCTCCGAGAATAATAATACCAG -3’ were used. For control, human GAPDH-specific sequences were amplified using the following primers: GAPDH forward 5’-ATGGGGAAGGTGAAGGTCGG-3’ and GAPDH reverse 5’-TTACTCCTTGGAGGCCATGTGG-3’. For RT-PCR reactions monitoring cDNA formation in in vivo experiments after *P.berghei* infection the following *P. berghei*-specific PCR primers were used: *eIF-5A* forward 5’-ATGTCAGACCACGAAACGT-3’/ *eIF5A* reverse 5’- TATGATGACATTTCTTTAAGC-3’ and *dhs* forward 5’-ATGGATGGGGTATTCAAAGA-3’/ *dhs* reverse 5’-CTAATCACTTTTTTCTCCTTTT-3’. To analyze the quality of the cellular total RNA *i* α-tubulin forward 5’-ATGAGAGAAGTAATAAGTAT-3’ and α-tubulin reverse 5’-TGTTGATAAAACTGAATTAT-3’ primers were applied, resulting in a specific α-tubulin fragment of 548 bp.

### Plasmodium transfection using shRNA expressing vectors

Parasite transfection using sh expression vectors without Pyrimethamine selection was performed as described in [24].

### Preparation of protein extracts from transfected *P. berghei* parasites

To detect eIF-5A and DHS expression in transfected and wildtype *P. berghei* parasites, intraerythrocytic stages were purified by CF11 Cellulose (Whatman) (Millipore, Schwalbach, Germany) to remove platelets and leukocytes. Parasites were lysed in 0.2% saponin and resuspended in PBS (LifeTechnologies/Invitrogen, Karlsruhe, (Germany). After determination of the protein concentration by Bradford assay [34], extracts were adjusted to the same protein concentration (20 μg) with PBS. Alternatively, for the detection of iNos protein, serum was applied from whole blood without anticoagulant according to a protocol from Proimmune [35].

### Western blot analysis

Western blots were performed using the i-Blot dry blotting device system from Invitrogen (Karlsruhe, Germany) for 5 min at 5.5 amp and 25 V. Protein extracts from blood stages of transfected parasites were resuspended in 1-fold Nupage buffer (Invitrogen, Karlsruhe, Germany) boiled and loaded onto a 12% SDS-polyacrylamide gel. Immunodetection was performed according to the protocol from the immunodetection kit from Amersham (Munich, Germany). Polyclonal anti-eIF5A antibodies (Eurogentec, Cologne, Germany) raised against the eIF-5A from *P. vivax* and anti-DHS antibodies against *P. falciparum* DHS were applied in dilutions of 1:1000 and 1:5000, respectively. Previous results had shown that the human DHS protein cross-reacts with the *P. berghei* DHS protein due to highly conserved regions and an overall amino acid identity of 56% (see within the results section) [11]. Dilutions of 1:1000 and 1:5000 of the antibody raised against the eIF-5A from *P. vivax* were used, since both proteins i.e. eIF-5A from *P. vivax* and *P. berghei,* share 97% amino acid identity [11].

### Induction of HeLa, jurkat and monomac cells and preparation of protein extracts

HeLa, Jurkat and Monomac were maintained in Dulbecco’s modified Eagle medium (DMEM) (Sigma Aldrich, Munich, Germany), supplemented with 10% (v/v) fetal bovine serum (FBS), L-glutamine (2 mM), sodium pyruvate (1 mM) penicillin (50000 U/ml) and streptomycin (5 mg/ml)).

For stress induction HeLa, Jurkat or Monomac cells were grown in overnight cultures under starving conditions (i.e. 1% FCS-containing medium). Thereafter, culture supernatants were substituted by DMEM containing 10% FCS and cells were further incubated for 3 hours. Finally, cell cultures were exposed for 4 hours to 50 ng/mL phorbol 12-myristate 13-acetate (PMA) and 1 μM of the calcium ionophore ionomycin. Subsequently, the respective cell cultures were washed several times with PBS.

In total 10^6^-10^7^ cells were lysed with CelLytic M solution (Sigma Aldrich, Munich, Germany) for 15 minutes on a rocker platform. The lysed cells were centrifuged at 12,000-20,000 x g to pellet the cellular debris. The supernatant, containing the cell lysate, were used for further analysis.

#### Determination of total nitric oxide

Concentrations of nitric oxide were determined by colorimetric detection according to the kit protocol from Enzo Life Sciences. Nitric oxide is converted to nitrate which is reduced to nitrite by the enzyme nitrate reductase followed by the colorimetric detection of nitrite as a coloured azo dye product which absorbs visible light at 560 nm. The determination allows the determination of both nitric oxide products nitrate and nitrite.

## Competing interests

The authors declare that they have no competing interests.

## Authors’ contributions

MK was involved in the transfection experiments, AS was responsible for the RT-PCR and Western Blot experiments. AKM and CHS performed the *P. berghei* transfection. BAM and TB were involved in the cloning of siRNA oligonucleotides. AK participated practically in the colorimetric assays and Western Blot experiments, prepared the manuscript and organized financial support, AKM and JH critically appraised the manuscript. We thank Barbara Langer for excellent technical assistance. All authors read and approved the final manuscript.

## Supplementary Material

Additional file 1 **Figure S1.**Unsuccessful silencing of parasitic EIF-5A by RNAi in 293T cells and subsequent monitoring by RT-PCR. A cotransfection was performed with: lane 1) EIF-5A-shRNA construct P# 5; lane 2) EIF-5A-shRNA construct P#; lane 3) EIF-5A-shRNA construct P# 7; lane 4) pcDNA3 based plasmodal EIF-5A expression vector; lane 5) *P. falciparum* eIF-5A expression vector and aquarin-5 specific siRNA; lane 6) EIF-5A-shRNA construct P# 18. Click here for file
