# Supplementary figures and images for: In vitro and in vivo silencing of plasmodial dhs and eIf-5a genes in a putative, non-canonical RNAi-related pathway
Source: BMC Microbiol. 2012 Jun 13;12:107. doi: 10.1186/1471-2180-12-107 (PMC3438091; doi:10.1186/1471-2180-12-107)

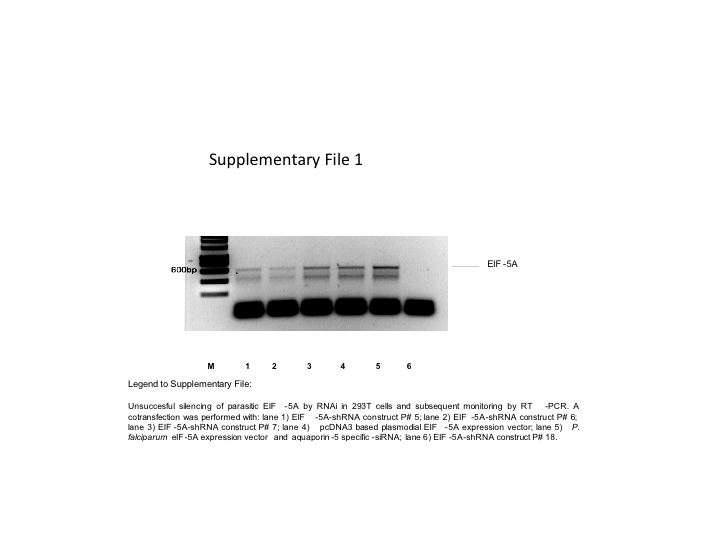

Supplement: Additional file 1 — Figure S1.Unsuccessful silencing of parasitic EIF-5A by RNAi in 293T cells and subsequent monitoring by RT-PCR. A cotransfection was performed with: lane 1) EIF-5A-shRNA construct P# 5; lane 2) EIF-5A-shRNA construct P#; lane 3) EIF-5A-shRNA construct P# 7; lane 4) pcDNA3 based plasmodal EIF-5A expression vector; lane 5) P. falciparum eIF-5A expression vector and aquarin-5 specific siRNA; lane 6) EIF-5A-shRNA construct P# 18. [file 1471-2180-12-107-S1.jpeg]
